# Role of Extracellular Vesicles in the Progression of Brain Tumors

**DOI:** 10.3390/biology13080586

**Published:** 2024-08-02

**Authors:** Gabriella Schiera, Carlo Maria Di Liegro, Francesco Vento, Italia Di Liegro

**Affiliations:** 1 Department of Biological, Chemical and Pharmaceutical Sciences and Technologies, University of Palermo, 90128 Palermo, Italy; carlomaria.diliegro@unipa.it; 2 Department of Biomedicine, Neurosciences and Advanced Diagnostics, University of Palermo, 90127 Palermo, Italy; francesco.vento01@community.unipa.it

**Keywords:** brain tumors, glioblastoma, extracellular vesicles (EVs), liquid biopsy, tumor biomarkers, drug nanocarriers

## Abstract

**Simple Summary:**

Brain tumors are among the most aggressive and difficult to cope with cancers among the many kinds which can affect the human body. These properties are due to their heterogeneity, and also to the fact that they are generated inside the brain, where they are isolated by the presence of the blood–brain barrier (BBB). However, like most cancer cells, they release extracellular vesicles (EVs), membrane-bound structures that transport many classes of molecules, involved in the growth and expansion of the cancer itself. EVs can cross the BBB in both directions; thus, they are now intensely studied because researchers hope that non-invasive isolation of EVs from the circulation may allow identification of cancer-specific biomarkers, which may be of help for the diagnosis as well as for envisaging the most useful therapy. Moreover, EVs themselves might also be used as a new type of carrier for delivering to the brain cancer-specific drugs.

**Abstract:**

Brain tumors, and, in particular, glioblastoma (GBM), are among the most aggressive forms of cancer. In spite of the advancement in the available therapies, both diagnosis and treatments are still unable to ensure pathology-free survival of the GBM patients for more than 12–15 months. At the basis of the still poor ability to cope with brain tumors, we can consider: (i) intra-tumor heterogeneity; (ii) heterogeneity of the tumor properties when we compare different patients; (iii) the blood–brain barrier (BBB), which makes difficult both isolation of tumor-specific biomarkers and delivering of therapeutic drugs to the brain. Recently, it is becoming increasingly clear that cancer cells release large amounts of extracellular vesicles (EVs) that transport metabolites, proteins, different classes of RNAs, DNA, and lipids. These structures are involved in the pathological process and characterize any particular form of cancer. Moreover, EVs are able to cross the BBB in both directions. Starting from these observations, researchers are now evaluating the possibility to use EVs purified from organic fluids (first of all, blood and saliva), in order to obtain, through non-invasive methods (liquid biopsy), tumor biomarkers, and, perhaps, also for obtaining nanocarriers for the targeted delivering of drugs.

## 1. Introduction

Extracellular vesicles (EVs) are membrane-bound structures, probably released from all cell types, in both prokaryotic and eukaryotic organisms [1,2,3]. These structures were initially considered a way used by cells to discard useless or even deleterious material; however, it has been then found that, besides this function, EVs play a fundamental role in cell-to-cell communications, as they deliver specific cargoes to the extracellular environment, from where they can also reach surrounding cells (target cells). The importance of EVs lies in their ability to transfer information to other cells, thus influencing their behavior. EVs indeed contain proteins, lipids, nucleic acids, sugars, and different metabolites, which can either act on neighboring cells as cellular signals or directly modify their metabolic activities. Moreover, the presence in EVs of the phospholipid bilayer can protect the cargo while also allowing the simultaneous release of multiple messengers. 

Over the course of evolution, both prokaryotes and eukaryotes have developed this elegant method of intercellular communication, which has allowed bacteria, for example, to coordinate the activities of their colony, monitor the environment, and influence the behavior of other bacteria, in a process known as quorum sensing [4,5,6,7] that also involves regulation of transcriptional activity in relation to the density of the colony. These strategies are also active in multicellular organisms and contribute in helping them to behave as a single, integrated system. Furthermore, it has been discovered that EVs are also involved in many interactions between prokaryotic and eukaryotic cells, as occurs, for example, at the intestinal level, between enterocytes and the resident microbiota [8,9].

## 2. History

EVs were found somewhat simultaneously in various physiological contexts; however, it was not immediately realized that this form of communication is a universally shared cellular property. In particular, EVs were originally observed, in 1946, by Chargaff and West in the plasma, as platelet-derived particles able to be involved in coagulant activity [10]; and were then defined “platelet dust” by Wolf in 1967 [11]. Among the first observations, there were also matrix vesicles, identified during bone calcification [12].

During the 1970s and 1980s, various experiments independently highlighted the release of vesicles from the plasma membrane of the microvilli of a rectal adenoma [13]. Various reports also focused on virus-like particles in human cell cultures and bovine serum [14], and on human prostatic cells that were found to release vesicles, later called prostasomes [15]. Around the same time, the first observations of membrane fragments originating from tumors were made [16,17].

Later, through ultrastructural studies, it was demonstrated that a class of vesicles are released from the intracellular multivesicular bodies (MVBs), and fuse with the plasma membrane, during differentiation of immature erythrocytes [18].

About ten years later, Raposo and colleagues [19] demonstrated that these vesicles, then called exosomes, isolated from B cells, transformed by Epstein Barr virus, contained antigens and were capable of inducing T cell responses.

In 2006–2007, with the discovery that they contain RNA (including microRNAs), EVs acquired further interest as mediators of intercellular communication in both physiological and pathological contexts [20]. As mentioned above, EVs have been then isolated from most cell types, including brain cells. Moreover, they have been found in body fluids, such as saliva, urine, nasal and bronchial lavage fluid, amniotic fluid, breast milk, plasma, serum, and seminal fluid [21]. This latter observation, as discussed in this review, has given a contribution to the possibility to set non-invasive diagnostic methods, described as “liquid biopsy” [22,23,24,25].

## 3. Extracellular Vesicles at the Basis of Intercellular Communication

EV size, content, and dimensions are highly heterogeneous and dynamic and depend on the cellular state and environmental conditions. Currently, at least three main subgroups of extracellular vesicles have been defined: (i) apoptotic bodies, that are released when vesiculation (blebbing) of the plasma membrane occurs during apoptosis; (ii) cellular microparticles/microvesicles/ectosomes, that are vesicles of different sizes that bud directly from the plasma membrane and are released into the extracellular environment; (iii) exosomes, that are intraluminal vesicles (ILVs) contained in the MVBs, which are released into the extracellular environment after the fusion of the MVBs with the membrane [21,26,27,28]. Despite the numerous studies conducted to date on the content, biogenesis, and secretion mechanisms of EVs, specific markers to distinguish the various classes of vesicles are not yet known.

Furthermore, it should be noted that the EV fraction obtained with the most popular isolation methods also includes other vesicular structures such as: (i) exosomal-like vesicles: they have a common origin with exosomes, but lack lipid raft microdomains and also vary in size; (ii) membrane particles: they have a diameter of 50–80 nm and come from the plasma membrane; (iii) extracellular membrane structures: also include linear or wrapped membrane fragments (derived, for example, from necrotic death) as well as lysosomal and cytoskeletal structures (nanotubules). The aforementioned particles, together with microparticles, microvesicles, nanoparticles, and oncosomes, are all indicated with the single name of ectosomes. This latter term emphasizes the fact that these vesicles have a cell outer membrane origin and are distinct from exosomes, which have an intracellular origin [28,29]. Ectosomes are very heterogeneous, both in size and composition; this variability arises from the fact that the membranes and contents of ectosomes are not identical to those of the plasma membrane and the cytosol of the cell of origin. Another important aspect of research on EVs concerns, indeed, the mechanisms that allow specific targeting of different molecules to EVs. The generation of ectosomes is very complex; some domains assemble along the plasma membrane; proteins destined to appear in ectosomes are sorted towards these domains, while proteins destined to remain in the cell are excluded. Concomitantly, specific cytosolic proteins and nucleic acids (mRNA and miRNA) accumulate in contact with plasma membrane domains. This mechanism is very similar to that of the budding of retroviruses (RNA viruses), and there is probably an evolutionary link between the two phenomena.

Trams’ research group described exosomes as “exfoliated vesicles with ecto-enzymatic activity” [30]. This work was followed by a report in which the release of small vesicles and tubules from rat reticulocytes was described [31]; in particular, with an electron microscopic study, the exocytosis of vesicular bodies of approximately 50 nm was evidenced. These structures were vesicles surrounded by a phospholipid bilayer (approximately 50–100 nm in diameter), and their size approximately overlapped that of viruses. Exosomes, observed for the first time in tumor and immune system cells, are released either constitutively or after stimulation. They act on the surrounding cells through direct contact, via endocytosis of the vesicles or by fusion between the vesicular membrane and the plasma membrane. Among the molecules transferred, there are mRNAs, miRNAs, oncogenic receptors, and viral particles. On the external surface of exosomes there is phosphatidylserine, and components such as the tetraspanin clusters of differentiation (CDs), including CD63, CD81, CD9, as well as the so-called lysosomal-associated membrane protein 1 (LAMP1). It is possible to isolate exosomes through differential centrifugation and subsequent ultracentrifugation in a sucrose gradient, and they can be analyzed by transmission electron microscopy (TEM), Western blot, and mass spectroscopy.

On the other hand, the microvesicles (MVs) were first described, as mentioned above, by Chargaff and West [10] in 1946, as factors with the potential to generate thrombin, which precipitated in platelet-free plasma. MVs are larger than exosomes (their diameter varying from 100 to 1000 nm) and are similar to those released from bacteria; MVs are generated by budding from the plasma membrane after stimulation, at low level; on the other hand, tumor cells released MVs in large amounts, and in a constitutive manner. The regulated release of MVs is effectively induced through the activation of receptors on the cell surface or following apoptotic processes, in which the intracellular concentration of Ca^2+^ ions increases. They were first observed as products of platelets, red blood cells, and endothelial cells. Besides their procoagulant role, activities in which their involvement has been demonstrated are: secretion of IL1β; pathogenesis of rheumatoid arthritis; pro-invasive role of tumors; induction of oncogenic transformation of cells; feto-maternal communication. MVs, like exosomes, also expose phosphatidylserine in the external layer of the membrane that surrounds them. Routine isolation and analytical methods include differential centrifugation and flow cytometry (FC).

The first researcher to use the term “apoptotic body” was Kerr in 1972 [32]. The subsequent foundational study on apoptosis was conducted by Sulston and Horvitz [33], who studied the lineage of the nematode *Caenorhabditis elegans*. Apoptotic bodies are 1–5 μm in diameter (similar to the size of platelets) and are released as bubbles in cells undergoing apoptotic death. They are characterized by externalization of phosphatidylserine into the membrane and may contain DNA fragments inside them. It has been found that apoptotic bodies can transfer oncogenes and DNA to surrounding cells [29]. 

Interestingly, as mentioned above, EVs are also produced by both Gram-negative and Gram-positive bacteria [34]. Gram-negative bacteria have 2 phospholipid membranes: the inner (cytoplasmic) membrane is the main diffusion barrier, but the cells are protected by the additional cell wall made up of peptidoglycan (in the periplasm and in the outer membrane). Unlike Gram-negative bacteria, Gram-positive bacteria are surrounded by a single membrane. Although the mechanism of vesicle formation is currently unclear, the membrane and lumen of Gram-positive vesicles are thought to arise from the cytoplasmic membrane and the cytoplasm, respectively. On the other hand, the external membrane is the interface with which Gram-negative bacteria interact with their environment, and, among the glycolipids that compose it, there are fats with extensive modifications to the carbohydrate component, the so-called lipopolysaccharides (LPSs). The EVs released from these bacteria, produced through external membrane budding, have a diameter between 20 and 250 nm. The molecular mechanism of vesiculation in bacteria is not completely known, but appears to be evolutionarily conserved in Gram-negative bacteria [21]. Through the release of vesicles, bacteria can also release a large amount of long-distance effector molecules protected by the EV cell membrane. The transfer can be directed towards animal cells, plant cells, or other bacteria. Bacterial EVs also contain molecules that mediate stress responses, formation of biofilms, and, in general, the influence exerted on the host [21].

## 4. Contents of Extracellular Vesicles

The content of extracellular vesicles represents a complex topic, not yet fully explored; however, current studies recognize that four large classes of molecules are contained within them: proteins, RNA, DNA, and lipids [35]. Variability does not only concern the type of molecules but also their quantity and distribution (either in the lumen or on the surface). Such properties, in turn, depend on the cell type and the microenvironment from which the exosomes draw origin.

In addition, surface marker proteins are commonly found in exosomes, among which are tetraspanins (CD9, CD63, CD81, CD82); these latter molecules, however, are no longer considered specific to exosomes because they have also been found in other EVs [36]. It must be highlighted that, to date, there is no single marker capable of univocally identifying a class of EVs, although there are groups of proteins that characterize some sub-groups of EVs.

Other highly represented proteins are heat shock proteins (i.e., Hsp70, and Hsp90) [37,38,39,40], membrane proteins, and proteins involved in membrane fusion and in transport (annexins, GTPases, flotillins). In exosomes isolated from the cerebrospinal fluid (CSF), researchers have found proteins typical of the central nervous system, among which are microglial cell markers (CD11 and CD45), markers of the nerve cells, and the apolipoprotein E (Apo E), typical of neurodegenerative pathologies [41,42].

Lipids, such as glycosphingolipids, cholesterol, phosphatidylserine, and ceramides, and proteins present in exosomes are incorporated into them during budding of the internal membrane, and are therefore common to the progenitor cell [43].

Exosomes deriving from brain cells contain high amounts of ceramide, which is involved in the budding of exosomes, in the lumen of multivesicular bodies [44]. Furthermore, a heterogeneous mixture of genetic material (mRNA, microRNA, and mitochondrial DNA) was also found in all kinds of EVs. 

EVs from mast cells and glioblastoma cells have been shown to carry and deliver functional messenger RNAs (mRNAs) [20,35,45]. The analysis carried out by the Lotvall’s group [20] identified approximately 1300 different mRNAs in exosomes from mast cells; furthermore, isolated polyadenylated mRNAs have been shown to be stable and functional in recipient cells, thus confirming that exosomes can act as mediators of the horizontal transfer of genetic information. Exosomal mRNAs, released by tumor cells, have been found to promote tumor growth and progression [46]. Furthermore, it has been demonstrated that human A375 melanoma cells produce the H1.0 histone protein and excrete it together with its mRNA via EVs [47]. Small RNAs have been found in human plasma, exosomes from saliva and breast milk; furthermore, vesicles released from human mesenchymal stem cells and human tracheobronchial epithelial cells were also found to contain small RNA species. Considering the pleiotropic properties attributed to small RNAs in recent years, great efforts have been focused on the identification of these small exosomal RNAs, in order to discover their biological functions in cell-to-cell communication, also with the aim to possibly use them as new biomarkers [48]. In particular, microRNAs have attracted greater interest, thanks to their potential role in regulating gene expression. Although the presence of small RNAs in exosomes was found for the first time in 2007 [20], only in 2012 it was clearly shown that miRNAs transferred by exosomes can have fundamental effects on dendritic target cells [49], where they can repress translation of specific target mRNAs. It is worth noting that the miRNA species transferred by exosomes does not reflect the miRNA repertoire of the cells of origin; thus, some miRNAs, regardless of cellular quantity, are selectively exported or retained within cells. All these observations suggest that loading of RNAs into exosomes is driven by specific choice mechanisms. Data obtained from “The miRNA registry” indicated the existence of over 2000 human miRNAs that can influence gene regulation of essential biological pathways, such as cell development, proliferation, apoptosis, cell signaling, and disease progression [50]. By simultaneously obtaining the enrichment of the exosomal fraction, and the isolation of the therein-contained RNAs, it has been demonstrated that the specific profile of RNAs in the exosomes can be used as a predictor of the response to treatment in patients suffering from specific pathologies, such as glioblastoma [51].

Although in smaller quantities than small RNAs, long non-coding RNAs (>200 nt: lncRNA) have also been identified in exosomes released from different cell types. LncRNAs are now recognized as important epigenetic regulators and exert this role with different molecular mechanisms, being able to act at both transcriptional and post-transcriptional levels, as well as to interact with proteins, RNA and DNA. Their possible oncogenic role has been demonstrated in tumors where de-regulated lncRNAs have been found; in particular, given their role as “miRNA sponges”, they could sequester miRNAs with tumor-suppressing activity, and prevent the degradation of mRNAs encoding oncogenic proteins [52].

Unlike RNAs, the presence of DNA in EVs has been much less studied, despite the initial discovery of the presence of oncogenic DNA in apoptotic bodies. The following species were detected in EVs: mitochondrial DNA (mtDNA) [53,54,55], single-stranded (ssDNA) and double-stranded DNA (dsDNA) [56], and amplified oncogenes (for example, c-Myc). Migration of mtDNA can occur via EVs, which may represent an alternative pathway through which altered mtDNA can enter other cells, promoting the spread of various pathologies. The presence of dsDNA, which represents genomic DNA, was highlighted in EVs and can give information on the mutations present in the tumor cells from which EVs derive. Although its function is still unknown, DNA in vesicles could be an important biomarker for tumors because it can allow to identify mutations present in parental tumor cells [57]. Even if the molecular mechanisms by which the genetic material appears in exosomes are not yet perfectly clear, databases have been created whose purpose is precisely to summarize the collection of molecules that make up exosomes. Among the databases from which it is possible to obtain useful information regarding the molecules involved in vesicle biogenesis, and the possible markers of pathologies, two of interest are Exocarta (http://www.exocarta.org, accessed on 1 June 2024) and Vesiclepedia (http://www.microvesicles.org, accessed on 1 June 2024) [58].

**Figure 1:**
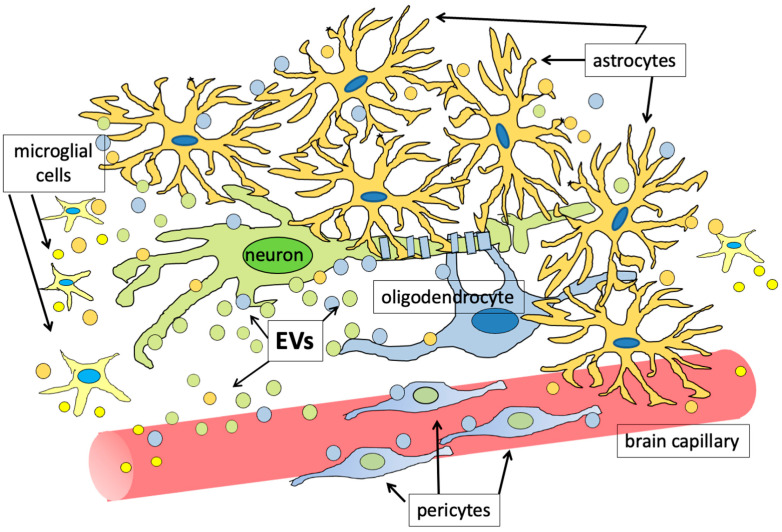
All brain cells release and receive extracellular vesicles (EVs), that seem to have fundamental roles in integrating both metabolic and cognitive functions. EVs have been represented as both smaller and larger vesicles because all the brain cells seem to release both exosomes and microvesicles (also called ectosomes). For clarity, EVs released from specific cytotypes have been represented in the same color as the releasing cells.

EVs have also been shown to carry important soluble mediators, such as cytokines. One of the first examples of the involvement of EVs in cytokine transport concerned interleukin 1β (IL-1β) [59]. Other examples of EV-associated cytokines were macrophage migration inhibitory factor (MIF) [60,61,62] and IL-32 [63]. Tumor necrosis factor (TNF) was also found to be secreted from microglial cells through EVs [64]; on the other hand, mast cells were found to release IL-6 into the vesicles following stimulation by IL-1 [65]. Similarly, in the retina it was found that the formation of the micro-vasculature requires a pericyte–endothelial interaction, mediated by factors, among which are the vascular endothelial growth factor (VEGF) and the platelet-derived growth factor subunit B (PDGFB), which are released into EVs [66]. It has been observed that VEGF is also present in the EVs produced by cancer patients [67]. Astrocytes produce FGF2 and VEGF together with integrin β1, a membrane protein, and are able to secrete them, at least in part, via EVs [68]. This latter study arose from the setting up of a co-culture system based on three brain cell types, in which neurons and astrocytes worked synergistically in order to induce the endothelial cells of the cerebral capillaries to form in vitro a monolayer with permeability properties similar to those of the physiological blood–brain barrier (BBB). These experiments allowed to demonstrate that astrocytes not only form direct contacts with the brain capillary endothelial cells (BCECs) through their feet but also release, through EVs, the aforementioned soluble factors in order to communicate with neurons and BCECs. The same group had previously demonstrated that also primary neurons in culture were capable of releasing EVs [69]. The ability to release extracellular vesicles adds new strategies to neuronal communication skills: one might assume that, in addition to interacting with target cells via neurotransmitters and neuromodulators at the synaptic level, they can modulate their survival and functions through the EV-mediated release of growth factors, which can act on the neurons themselves or influence differentiation and functioning of other cell types, first and foremost astrocytes and endothelial cells. Factors present in EVs could guarantee protection to the factors in the extracellular matrix (ECM) and/or modulate their release towards the target cells. Furthermore, in contrast to classical neurotransmission, it was possible to observe how EVs arise from different sites of the neuron, in particular from the cell body and from neurites [69,70]. The fact that both neurons and glial cells release EVs, in both physiological and pathological conditions, offers the opportunity of exploring new ways in which these cells communicate, even over long distances, with each other, and with the rest of the organism. It has also been hypothesized that this mechanism could be the basis of phenomena such as learning and memory: for over 50 years, the causal relationship between the aforementioned processes and the ability of neuronal circuits to undergo long-term adaptations between their connections has being studied; although there are still many shadows to resolve, it is now quite clear that there is an intense cross-talk between neurons and the non-neuronal cells that surround them. Moreover, this cross-talk can be largely mediated by EVs; in particular, astrocytes are supposed to play a crucial role in the formation and in the strengthening of neuronal circuits, and many of the active molecules released from them are probably loaded into EVs [70,71]. In Figure 1, the ability of all brain cells to exchange information through EVs, in physiological conditions, is schematically shown.

A further interesting hypothesis concerning EVs suggests that the RNAs present in them can also function as protein transporters [35,47]; in other words, thanks to their ability to interact with RNAs, RNA-binding proteins (RBPs) could enter EVs and thus reach surrounding cells, where, if also able to bind to DNA, they could modify chromatin structure and/or the transcriptional activity [35], assuming a leading role in the epigenetic action induced by the EV cargo in recipient cells. This hypothesis arose from the observation that many RBPs are capable of binding both RNA and DNA. Among these proteins, we can consider: (i) the histones themselves; (ii) proteins containing the cold-shock domain, such as the cold-shock domain-containing protein C2 (CSD-C2), also known as PIPPin [72], and Y-box-binding protein 1 (YB-1), a transcription factor, that is also a component of mRNA–protein complexes (mRNPs) [73]; (iii) proteins containing zinc finger domains, that, besides DNA, can also bind RNA. Interestingly, in addition to all these proteins, it has been found that some proteins that lack conventional nucleic acid binding motifs might also be considered in the hypothesis; for example, SOD 1, the metalloenzyme known for its catalytic action on the superoxide anion, also plays an important role as a transcription factor, and as an RNA-binding protein (RBP) [74]. Actually, based on exosome proteomics, the presence of several transcriptional regulators in EVs was clearly shown [75]. Some proteins have direct and known transcriptional activity, while others can be considered as multifunctional factors [75]. For example, the Myelin expression factor 2 (MYEF-2), already known as a transcriptional repressor of the gene for the myelin basic protein, was also found in melanoma cell-derived EVs, where it is bound to the H1.0 histone mRNA [47]. Thus, some EV-transported DNA- or chromatin-binding proteins may also behave like RBPs. As a result, in these cases, RNA is used as a Trojan horse to allow these proteins to reach the recipient cells, where they can then detach from RNA, and bind to chromatin at the level of specific genes, thus modifying their expression. Of course, further studies will be necessary to provide further confirmation of this innovative hypothesis.

In the brain, in addition to their physiological roles, EVs are clearly involved in pathological processes, and, in particular, in cerebral cancer. MVs released from G2624 oligodendroglioma cells, when added to primary cultures of rat neurons and astrocytes, are able to block neuritic growth and can induce apoptotic death in a dose-dependent manner. Western analysis of vesicles shed from G26/24 cells evidenced the presence of TRAIL and Fas-L, two factors that can cooperate in inducing brain cell death [37,76].

## 5. Gliomas

Gliomas are the main brain tumors. Histologically, they still have some characteristics normally found in glial cells and are therefore named on the basis of these similarities. However, it is still under investigation whether gliomas arise from normal glial cells, from glial or neural precursor stem cells, or from other cell types [77]. Historically, the diagnosis and classification of these tumors were purely histological in nature. According to classification of the World Health Organization (WHO) published in 2007, the main groups of glial tumors consist of astrocytic, oligodendroglial, oligoastrocytic, ependymal, neuronal, and mixed glio-neuronal (such as gangliogliomas) tumors [78]. The standard criteria used by pathologists for diagnosis and staging of gliomas, as mentioned, were defined by the WHO 2007 classification [78]. The WHO classification was based on histological diagnosis and staging, in order to define the degree of malignancy; thus, the tumors are classified from grades I to IV, generally on the basis of increased malignancy, cellular atypia, mitotic activity, also considering, for some cancer cell subtypes, the possibility to include microvascular proliferation and/or necrosis levels [79]. Since this histological classification actually has limitations, a further revision was made. One of the problems associated to an exclusively histological diagnosis is the significant variability found when comparing interpretation from different observers. Although several studies on the matter arrived at different conclusions, it was actually found that agreement on diagnosis for one patient, in a group of neuropathologists, can even go down to 52%, with profound discrepancies when distinguishing among astrocytic, oligoastrocytic, and other oligodendroglioma types, as well as between II and III degree tumors [80]. Diagnostic criteria for most tumors may indeed result to be technically imprecise, because of the tumor intrinsic heterogeneity [81]. Furthermore, accurate histological classification could be further complicated by the insufficiency and low representativeness of tissue samples [80]. These problems, together with the impact of the discoveries relating to the molecular profiles of gliomas, finally led the WHO to revise, in 2016, the Classification of Tumors of the Nervous Central System, well before the expected date [82]. One of the fundamental changes was the inclusion of molecular diagnostic criteria among those used for the classification of infiltrating gliomas. In addition to the histological subtypes of the previous classification (astrocytoma, oligodendroglioma, and oligoastrocytoma), the new criteria involve the evaluation of mutations, such as those found in the gene encoding the isocitrate dehydrogenase 1 (IDH1), the 1p/19q co-deletion, and some histone mutations. Thus, astrocytoma and GBM are divided into IDH-mutated and wild-type IDH, oligodendrogliomas in IDH-mutated and tumors with 1p/19q co-deletion, and, finally, diffuse midline gliomas are defined by the histone H3K27M mutation [83,84,85]. In case of impossibility to carry out molecular tests, the tumors are classified on the basis of what was indicated by the 2007 classification, only based on histology (astrocytoma, oligodendroglioma, and oligoastrocytoma), but the word “NOS” (Not Otherwise Specified) is added in order to indicate the absence of the additional molecular diagnostic criteria [82]. An updated version for the classification of brain tumors was recently published, which allows the classification of new types of tumors, based on differences in DNA methylation (DNA methylome profiling) [86]; all these criteria are aimed at providing accurate guidance to all pathologists in the world, for the purpose of a diagnosis as accurate as possible. All the molecular information, in any case, must be appropriately integrated, case by case, with the clinical, imaging and histological context, in order to delineate the unique properties of a single tumor at best; this kind of approach is also in line with the ever-growing tendency to “individualization” of therapeutic plans, especially in the oncological field. Actually, as discussed below, purification of extracellular vesicles from organic fluids may offer a new non-invasive approach for analyzing biomarkers able to give more precise information on the specific properties of the patient’s brain cancer.

## 6. EVs and the Tumor Microenvironment

Since their discovery, it was clear that EVs are released in large amounts by cancer cells that use them to modify their environment. In the case of gliomas, EVs released from tumor cells as well as those derived from other cells present in the tumor microenvironment are involved in proliferation and invasion of cancer cells as well as in the transformation of surrounding cells they reach. These EV-mediated events result in the generation of a niche that supports tumor angiogenesis, immunosuppression, and the acquisition of increasingly malignant traits by tumor cells [87,88,89]. Probably, at the beginning of their evolutionary history, EVs were only involved in the elimination from cells of waste material. However, the ability to exchange a variety of molecules allowed, over time, to synchronize the activities of individual cells within the entire tissue population. On the other hand, the same ability can easily transform a physiological condition into a pathological one when cells use EVs to transfer molecules that somehow “infect” the surrounding ones and modify the extracellular environment, thus allowing the pathology spread. Some studies have shown that a single glioma cell can produce almost 10,000 EVs in about 48 h, which allow it to easily invade the surrounding brain parenchyma [90].

Thanks to in vitro studies and microarray analyses, glioma-derived exosomes have been shown to carry several miRNAs, among which are miR-21, miR-29a, miR-221, and miR-222, which can trigger proliferation and inhibit apoptosis of tumor cells [91,92]. EVs released from glioblastoma cells can transfer, for example, miR-21 and miR-451 to microglia and macrophages. In particular, it has been suggested that cells of the monocytic lineage, particularly affected by glioma cells, increased the production and secretion of cytokines, and the expression of the Membrane Type 1 Matrix Metalloproteinase (MT1-MMP); at the same time, the phagocytic capacity of macrophages is increased [93].

In general, the presence of different RNA species in EVs is one of the most important aspects of their functions. RNAs transferred from EVs can be taken up indeed by surrounding cells and induce profound changes in the gene expression of recipient cells: (i) mRNAs can be translated; (ii) miRNAs can bind endogenous mRNAs, thus inducing their degradation, or at least translation inhibition; (iii) lncRNAs can function as drivers for transcriptional factors, and as sponges for endogenous miRNAs, reducing the ability of these latter molecules to inhibit endogenous mRNAs [94]. In other words, all RNA species can act as epigenetic determinants, capable of modifying gene expression in recipient cells [35]. In the case of mRNAs, we can hypothesize that specific RBPs are also involved. Interestingly, it was also found that some microRNAs released from GBM and transported by EVs exhibit post-transcriptional modifications, such as uridylated 3’ ends; for example, the mature miR-451, one of the most actively secreted by glioblastoma, contains two uridine residues at the 3′ end [94]. These observations suggest that RBPs able to recognize U residues may be involved in the EV-mediated transport.

**Figure 2:**
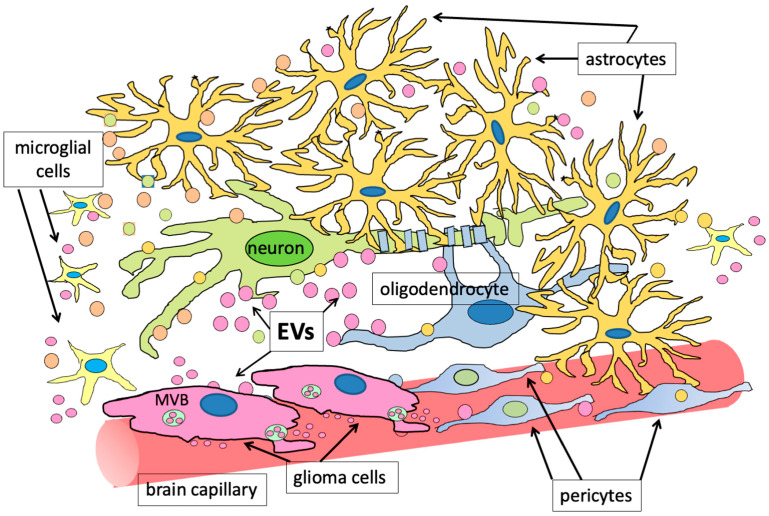
The figure represents the same brain microenvironment drawn in Figure 1. Here, however, glioma cells are also present. As discussed in the text, cancer cells sit on the vessels in order to have easy access to metabolites and oxygen. They release a high amount of EVs (pink in the figure): both exosomes from multivesicular bodies (MVB) and ectosomes, directly from the plasma membrane. The content of these EVs, when transferred to the neighboring cells, can induce a modification of their activities and, as a consequence, also a change in the content of the EVs released from these surrounding cells. In order to make evident this phenomenon, in the figure, most of the EVs released from the other cells have not been represented anymore with the same color as the producing cells, but in a new color (orange).

In the tumor microenvironment, different niches, each containing cells with different properties, have been evidenced: (i) perivascular, (ii) hypoxic, and (iii) invasive. The perivascular and the hypoxic niches characterize the tumor tissue and contain both necrotic tissue and aberrant blood vessels. On the other hand, the third niche is located at the interface between the tumor and the healthy brain parenchyma; in this region, glioblastoma stem cells (GSCs) adhere to normal vessels and induce endothelial cells to assume an aberrant behavior, and to invade the healthy tissue. Actually, GSCs can indeed either induce neovascularization, by releasing VEGF-containing EVs into their microenvironment, or can adhere to pre-existing vessels, giving rise to the process known as vessel co-option [95,96,97]. Interestingly, it seems that different subtypes of GSCs can induce different responses into the endothelial cells, thus causing angiogenesis or circumferential vascular growth, also called vasectasia; interestingly, it has been recently reported that this latter process can be triggered by EVs containing, and transferring to endothelial cells, the EGF receptor (EGFR) [98]. Actually, all the described events depend on a cross-talk between GSCs and other cells, and involve secretion of different factors, among which include TGFβ and FGF2, released by endothelial cells, that, together with VEGF, produced by GSCs, induce vessel modifications in the perivascular site [99]. The cancer cell-derived EVs are indeed able to induce modifications of the phenotype of the surrounding brain cells, among which are astrocytes, which start producing molecules that support further cancer growth (Figure 2) [100,101,102].

By sitting on vessels (thus forming a vascular niche), and migrating along them (invasive niche), tumor cells and, in particular, GSCs can find access to oxygen, and to the nutrients necessary for their metabolism and further development. However, this pathway also exposes tumor cells to difficulties, such as, for example, the need to adapt to confined spaces; for this reason, by drastically reducing their volumes and by competing with normal astrocytes and pericytes for vital space, they interact stably with the vessels at the level of the basal lamina and finally manage to migrate [99]. An interesting aspect of intratumoral heterogeneity is the existence in the tumor of non-mitotic territories, the genesis of which is still under study. As an example, a possible involvement of β-catenin in determining the antiproliferative behavior of these territories has been reported; in particular, it seems that, thanks to its ability to induce the production of miR-302, which targets cyclin D1, β-catenin reduces stem-like properties in tumor cells [103].

ECM remodeling mainly occurs through different steps, involving degradation of extracellular proteins, as well as their post-translational modifications. Some ECM receptors, such as integrins, mediate intercellular communication and exchange between tumor cells and their microenvironment. Metalloproteases significantly contribute to ECM remodeling, and it has been found that the levels of some of them (i.e., MMP-2 and MMP-9) increase as miRNA-26a passes from GSCs to endothelial cells, via EVs. Besides ECM degradation, as mentioned above, these events promote the induction of angiogenesis. Interestingly, in glioblastoma, the expression of the transmembrane glycoprotein CD44 relates to the tumor grade; moreover, glioma cell-derived EVs contain high levels of CD44 [104], thus allowing attraction of MMPs that promote ECM remodeling. Most of these events are mediated by the ability of the tumor cells to produce large amounts of EVs that indeed contain extracellular proteins, among which are the extracellular matrix protein 1 (ECM-1) and collagen IV, as well as ECM-remodeling enzymes, among which are gelatinase MMP-2 (both in pro-enzymatic and active form), and the pro-MMP9 [105,106,107,108]. 

These EVs also contain plasminogen activators, such as PA-PAI complexes, tissue PA (tPA), and PA urokinase (uPA), and tissue inhibitors of MMPs, such as TIMP1 and TIMP2, which contribute to the angiogenic activity related to tumor growth. Another protein, capable of acting as an ECM remodeling factor and present in GBM-derived EVs, is cathepsin D [109].

Tumor cells can also cause ECM remodeling indirectly; in vitro studies have indeed shown that EVs derived from glioma cells can induce adjacent cells (cancer-associated fibroblasts: CAFs) to secrete ECM components. Interestingly, Trylcova and colleagues [110] reported that CAF-conditioned medium has an effect on glioma cell migration in vitro; moreover, they found, by immunofluorescence, the presence of cells expressing CAF markers in tumor samples from patients; on the basis of these observations, the authors suggested a CAF-dependent ECM remodeling both in vivo and in culture. Moreover, EVs produced by GBM can induce overexpression of MT1-MMP in microglia, thus inducing these latter cells to support tumor growth. In addition, the Semaphorin3A (Sema3A), present on the surface of EVs, causes abnormal cell-substrate adhesion and loss of barrier integrity [111]. Furthermore, exosomes released under hypoxic conditions are rich in metalloproteases and lysyl oxidases and can therefore promote angiogenesis. The secreted protein, acidic and rich in cysteine (SPARC; also known as osteonectin), is a protein strongly expressed at the perivascular level by cells adjacent to the vessels; it modulates interactions between the cells and the extracellular matrix and promotes migration and invasion [112]. It is not yet known whether this protein is secreted through EVs from brain cancer cells; however, it has been found in EVs released, for example, by colon cancer cells [113].

Due to its proximity to blood vessels, the perivascular niche (PVN) is characterized by increased expression of angiogenesis-related genes, such as MMP9, the hyaluronan synthase 1 (HAS1), the vascular endothelial growth factor (VEGF), the platelet-derived growth factor receptor (PDGFR), the epidermal growth factor receptor (EGFR), and Tenascin C (TNC). Most of these overexpressed genes are part of pathways known to be altered in GBM. 

Actually, many cell types interact at the level of PVN, thus providing different potential EV-specific markers. For example, GSCs can differentiate into pericytes under TGF-β stimulation [114] and are able to recruit tumor-associated macrophages (TAMs, or M2), which promote tumor growth, by allowing immunoescaping. Other pathways include those engaging Wnt5, Notch, integrin α6, angiopoietin 2, L1 Cell Adhesion Molecule (L1CAM), Semaphorin 3C (SEMA3C), and bradykinin, all of which are involved in the survival, progression, and angiogenesis of GSCs. Hypoxia, and the hypoxia-inducible transcriptional factor (HIF), are, however, the main driving factors underlying the process of neoangiogenesis in gliomas. One of the genes activated by HIF-1α is that encoding VEGF, which is the main stimulator of endothelial cell proliferation, thus causing the growth of new vessels in the hypoxic regions [115]. However, HIF-1α and neo-angiogenesis can also be activated independently of hypoxia, through the activation of the Wnt/β-catenin pathway (promoters of c-Myc activation) [116].

Another factor known to stimulate both cell proliferation and migration is the epidermal growth factor (EGF); the overexpression of its receptor (EGFR) characterizes, indeed, high-grade gliomas [117]. The EGFR gene is amplified in 40% of cases of malignant gliomas, and about 50% of these cells have a mutated form of the receptor (EGFRvIII), which lacks the ligand-binding domain and is thus constitutively active. Through RNA sequencing, miRNA-21a was identified as a regulatory molecule, able to induce increased proliferation of neuronal stem cells (NSCs), by targeting the mRNAs encoding the SRY-box transcription factor 2 (SOX2), and the signal transducer and activator of transcription 3 (STAT3), thus contributing to the invasiveness of GBM. Other molecules identified in GBM cell-derived EVs include miR-9, miR-let7b, miR-124, and miR-137 [118]. In the same study [118], it was also discovered that GMCs overexpress VEGF when they are treated with exosomes enriched in miR-21a; given the already mentioned properties of VEGF, its overexpression in turn induces endothelial cells to form new vessels.

Very large amounts of EVs were isolated from the plasma of GBM patients, and it was found that they contain hypoxia-regulated proteins, such as, among others, matrix metalloprotease 9 (MMP9), platelet-derived growth factor (PDGF), interleukin-8 (IL-8), and caveolin 1 (CAV1). Similarly, high levels of Interleukin 8 and other hypoxia-induced cytokines were also found in exosomes from human GBM xenografts introduced into mice [119].

Furthermore, hypoxia has been shown to promote autophagy and M2 polarization of tumor-associated macrophages (TAMs) [119]. The underlying mechanism involves IL-6 (interleukin 6) and the activator of transcription (STAT). The facilitation of autophagy processes and macrophage polarization lead to an increase in the myeloid-derived suppressor cells (MDSCs) proliferation and migration, both in vitro and in vivo [120]. In particular, expansion of MDSCs seems to depend on miR-10a and miR-21, present in the hypoxic exosomes. It has also been recently demonstrated that the long non-coding RNA POU3F3 (lnc-POU3F3), transported by GBM EVs, can induce increased expression of EGFR and VEGFA, thus positively influencing angiogenesis [121]. It has also been found that, under hypoxic conditions, cancer cells release small extracellular vesicles able to induce GSCs to undergo a pericyte–phenotype transition, thus causing a failure of anti-angiogenic therapies [122]. Actually, it appears of central importance to better define the composition of EVs released from cancer cells and, in particular, from GSCs, in order to understand their role in the high level of heterogeneity that characterizes brain cancer and glioblastoma in particular. A recent paper allowed, for example, to suggest that different subtypes of GSCs are enriched in specific combinations of proteins, nucleic acids, lipids and metabolites, and that the different GSC subtypes might cooperate in determining the high complexity of the tumor as a whole [123]. Different mechanisms have been recently described for explaining the ability of glioblastoma to evade immune surveillance; for example, preliminary evidence has been reported that primary cilia allow release of IL-6, thus contributing to the recruitment of the immunosuppressive M2 subtype of macrophages [124]. In addition, it has been found that cyclooxygenase-2 (Cox-2), transferred by EVs, promotes M2 macrophage polarization too [125].

## 7. EVs in Immunomodulation

Glioma cells in the tumor microenvironment are surrounded by normal stromal cells, such as astrocytes and ependymal cells, oligodendrocytes, and microglia. By producing and releasing, at least in part by exosomes, immunomodulatory molecules, GBM cells can induce immunosuppressive functions in microglia [93,126]. These mechanisms are mediated by regulatory T cells, tumor-associated macrophages, and myeloid-derived suppressor cells (MDSCs). Microglia can induce or hinder proliferation of neuronal stem cells (NSCs), as well as differentiation and survival of neurons, through the secretion of cytokines capable of triggering various inflammatory processes in the brain. Furthermore, macrophages are key players in the development of a link between innate and adaptive immunity. Glioma cells are in a hypoxic state and produce miRNA-12, through which they induce macrophages to assume an M2 phenotype, via the STAT3 and NF-κB pathways. In glioma progression, M2 polarization is the most important event in modulating the microenvironmental immune system [127]. Microglial cells also release exosomes containing cytokines that support tumor growth. For example, tumor necrosis factor α (TNF-α) has also been found in them [64]. TNF-α and Interleukin-1 β (IL-1β) induce in glioma cells the production of the heat shock protein known as crystallin alpha B (CRYAB, also indicated as HspB5) [128]. Given the anti-apoptotic activity of this latter factor, survival of tumor cells in the microenvironment is thus favored. In some studies, neural stem cell-derived EVs (NSC-EVs) have been shown to mediate communication between the immune microenvironment and the neurogenic niche [128]. This EV-based cross-talk turns out to be two-sided. Specifically, the EVs generated by the components of the neurogenic niche are able to affect the cellular properties of glioma cells, especially in the very early stages of the disease. On the other hand, tumor cell-derived exosomes (TEXs) can influence the activity of immune system cells (IS). In the case of head and neck squamous cell carcinoma, for example, the exosomal “programmed death-ligand 1” (PD-L1) protein has been reported to be involved in this effect [129]. Natural killer (NK) cells are involved in immune surveillance phenomena, but it has been found that glioma cells release EVs that can mediate inhibition of the natural killer group 2D (NKG2D) cytotoxic lymphocyte receptor; due to this action, the antitumor activity of the NK cells is suppressed [130]. Dendritic cells (DCs) are involved in the activation of immune cells through the presentation of antigens. B cells are part of humoral immunity, and TEXs regulate B-Reg cell-mediated immunosuppression via PD-L1 in glioblastoma. Circulating TEXs influence the expression of costimulatory molecules for B cell activity. Suppressor cells (MDSCs), of myeloid derivation, are immunogenically immature cells; their differentiation and activity are inhibited by glioma cells that deliver to them, via EVs, a series of microRNAs, such as miRNA-10 (which acts via the RAR Related Orphan Receptor A, Rora), miRNA-21 (which acts via the phosphatase and tensin homolog, PTEN, oncosuppressor pathway), miRNA-29 (which acts via the HMG-Box transcription factor 1, Hbp1), and miRNA92 (which influences the pathway involving the protein kinase A regulatory subunit I alpha, PRKAR1A, pathway) [131]. In general, glioma cells are surrounded by a predominantly immunosuppressed environment, characterized by a low number of infiltrating lymphocytes, high concentrations of immunosuppressive cytokines such as TGFβ and IL-10 (released by brain stromal cells), and of indoleamine 2,3-dioxygenase 1 and tryptophan 2,3-dioxygenase 2 (IDO/TDO), both of which could stimulate the accumulation of regulatory T cells (Tregs). Actually, it is very important for immunosuppression that the already mentioned tumor-associated macrophages (TAMs) also communicate with other types of immune cells. Chemokines secreted by TAMs indeed contribute in reducing T cell infiltration, thus creating a fully immunosuppressed microenvironment [132]. Actually, factors secreted by glioblastoma cells activate aryl hydrocarbon receptor (AHR) in TAMs; activation of AHR, in turn, enhances the expression of the Krüppel-like factor 4 (KLF4), and suppresses NF-κB signaling by TAMs. The TAM ectonucleotidase CD39 is then upregulated and, in synergy with CD73, promotes CD8+ T cell dysfunction, through increased adenosine expression [133]. In a mouse model, tumor cells also induce mTOR expression in microglia, and mTOR-mediated STAT3 signaling contributes to the M2-like microglial phenotype, which impairs effector T cell infiltration, tumor proliferation, and immune responses [126]. IL6-activated STAT3 positively regulates the promoter of the B7 homolog 4 (B7-H4) gene, thus inducing an increase in the B7-H4 levels in TAMs; this step is essential for blocking the T cell immune responses [134]. TAMs also upregulate a variety of surface molecules that inhibit T cell activation and promote induction of T cell apoptosis, including CD80/CD86, CD95, CD70, and PD-L1. All these events, at least in part due to the EV-mediated release of both proteins and nucleic acids, activate tumor immunoescaping because they prevent the immune response against glioma by also decreasing the number of infiltrated effector cells [135,136,137,138].

Interestingly, it has been found that, when glioblastoma cells are cultured in 3D culture systems, the amount of extracellular vesicles released from these cells increases with respect to those produced by the same cells cultured in 2D systems. Moreover, these vesicles are enriched in signaling molecules that can potentiate tumor progression and immunosuppressive properties [139]. 

## 8. Formation of the Pre-Metastatic Niche

On the basis of the studies carried out so far, and as discussed above, we can say that exosomes derived from tumor cells significantly contribute to different aspects of the metastatic spread of the primary tumor, including invasion of surrounding tissues, angiogenesis, modulation of immune mechanisms, and formation of the pre-metastatic niche [102,140]. The roles played by exosomes in the formation of the pre-metastatic niche are different and range from metabolic reprogramming to the recruitment of numerous immune and non-immune stromal cells in order to facilitate metastatic growth.

Based on recent research, it is clear that metastatic disease is not a random process. The spread of tumor cells in secondary organs occurs in an organ-specific manner, depending on the type of cancer, and this phenomenon allowed Stephen Paget to propose the so-called “seed and soil” hypothesis [141]. This concept of metastatic growth specificity has been confirmed by the observation that, even when tumor cells can be found in the vasculature of multiple organs, only selective sites consistently develop metastatic deposits [142]. Actually, tumor cell spread to secondary organs is promoted by the preventive formation of suitable environments at distant sites, the so-called pre-metastatic niches.

The spread of tumor cells to secondary organs thus temporally follows the formation of a premetastatic niche, which represents a highly specialized area in distant sites from the main tumor. For example, it was shown that bone marrow-derived hematopoietic progenitor cells (BMDCs) can nest at specific sites that act as primers expressing the so-called very late antigen-4 (VLA-4), and recruiting tumor secretory factors, leading to the accumulation of the VLA-4 ligand fibronectin in pre-metastatic organs [143]. Since then, numerous studies have established that variable factors secreted by the tumor are responsible for the predisposition of a permissive secondary organ, through the modulation of the activity of various types of stromal cells [144].

All these studies have been mainly based on classical modes of cellular communication which include the release of cytokines, chemokines, and growth factors; however, it is becoming increasingly evident that, also in the case of metastatic spreading, non-canonical signaling pathways are also involved in the formation of pre-metastatic niches. It is also important to underline that metastases are responsible for 90% of cancer-related deaths, and that EVs are involved, as primary invading elements, in cancer spreading [145,146].

The evidence of EV involvement in all the stages of cancer development and spreading has stimulated more advanced examination of EV contents. In particular, the miRNA profile of glioma cell-derived exosomes has been analyzed. The results of these research studies have confirmed involvement of a variety of miRNAs in promoting cancer development and invasion, as well as establishment of a pro-tumor microenvironment [147,148,149,150,151]. It is also known that some miRNAs can act in the opposite way, inhibiting tumor growth [151,152,153]. Similarly, long non-coding RNAs (lncRNAs) can play a promoting role in cancer development and invasion [151,154,155,156,157].

When circulating tumor cells (CTCs) reach the brain, after crossing the BBB, the formation of the neurovascular niche is necessary to form brain metastases. In order to survive after extravasation, these cells require continuous contact with the ECM, and with the cerebral microvessels. Numerous studies have indeed demonstrated that CTCs bind to the basal lamina, thus ensuring to themselves access to oxygen and metabolites, as well as a position in the middle of vessels, which promotes metastatic growth [158]. Actually, indeed, the initial phase of migration into the brain parenchyma is frequently fatal for the CTCs [159].

CTCs can also form micrometastases that remain dormant in the perivascular microenvironment for many years [160]. Recent work shows that the CTC dormancy phase depends on endothelial-derived thrombospondin-1 and the laminin-211 deposited by astrocyte into the perivascular space [161]. While the precise mechanisms leading to awakening of dormant micrometastases remain unknown, metastatic progression can follow various invasion patterns. Interestingly, the properties of the primary tumor are not strong predictors of what pattern of invasion will be observed at the metastatic locus [162]. 

Metastatic growth in the brain takes place along the following patterns: (i) non-infiltrating growth, thus producing a tumor boundary without infiltration into the adjacent tissue; (ii) diffuse invasion, in which the brain parenchyma undergoes infiltration by either single cells or groups of them; (iii) vascular co-optation, in which tumor cells adhere to the blood vessels that penetrate into the brain tissue [163].

It is worth noting that the neurovascular niche, in brain tumors, is not only involved in metastasis but, first of all, in the invasion of brain parenchyma by the tumor cells that move along the cerebral blood vessels, and that also induce disruption of the blood–brain barrier [164]. Also in these processes, the role of EVs, which are able to cross the BBB, is determinant, even if the precise mechanisms involved are not yet completely clear [164,165,166].

The non-angiogenic process in which cancer cells spread along the blood vessels is called blood vessel co-optation. The mechanisms with which they implement this process include: (i) adhesion to pre-existing vessels without disruption of the BBB; (ii) an influence on pericytes and astrocytes, which in turn induce a modification of the endothelial cell functions, ECM remodeling, and, very often, BBB disruption. In addition, tumor cells are also capable of moving diffusely throughout the brain parenchyma. The ability of co-opting pre-existing vessels helps tumor cells to survive, ensuring their access to nutrients and oxygen, as well as providing them with a path for further invasions. Vessel co-option can also allow BBB escaping, as happens for acute lymphoblastic leukemia cells, that transit along the vessels, thanks to the α6 integrin, thus entering the cerebrospinal fluid without extravasating into the brain [97,167]. 

Preclinical evidence indicates that vessel co-option throughout the brain is a central step for brain invasion by metastatic cells from breast, lung, skin, and colorectal cancer. Interestingly, similar findings concern low-grade gliomas, where vascular co-option is a prominent feature of early malignant growth [168]. These observations have led to paying much attention when developing anti-angiogenic drugs which might target the pathological mechanisms underlying blood vessel growth. Regarding the mechanism of vessel co-option in brain metastases, there is an involvement of adhesion molecules present on the surface of tumor cells, including β1 integrin and L1CAM, which establish engraftment, that is, the formation of a coating along the surface of the vessel, and the creation of an integrated growth front, starting from the existing vascular system. In GBM, several factors promote blood vessel co-option, including bradykinin, breast-derived growth inhibitor, the CXC motif chemokine receptor 4 (CXCR4), EGFRvIII, Angiopoietin-2, IL8, the endoplasmic reticulum to nucleus signaling protein 1 (IRE-1α), and Wnt7 [169]. Vascular co-option may or may not compromise BBB function. When this happens, the GBMs behave like pericytes and start influencing the neurovascular system (this phenomenon is called “pericyte mimicry”) [170,171]. Interestingly, a pericyte-like spreading along the blood vessels was also observed in brain metastases from peripheral tumors [48]. Moreover, EVs have been found to also have a role in the formation of brain metastases [172,173].

## 9. EVs: The Tumor Biomarkers of the Future?

The main determinants that make a diagnostic marker ideal can be summarized by the following points: bioavailability, ease of isolation, and, finally, ability to accurately provide important information on the state of the disease. It is not surprising, therefore, that, over the last decade, tumor cell-derived EVs are being increasingly considered as a possible future diagnostic tool. Recently, great emphasis has been placed on the development and optimization of diagnostics through non-invasive (for example, urine and saliva samples) or minimally invasive (such as blood and cerebrospinal fluid) sampling because they offer many advantages over invasive forms of sampling. These approaches have been grouped under the term “liquid biopsy”, that indeed refers to the possibility to identify biomarkers in body fluids [92,174,175,176].

In the case of brain tumors, this is also possible given the already mentioned ability of EVs to cross the BBB in both directions. EV-based advantages include reduced patient discomfort, greater speed, and lower analysis costs. Among the components of the exosomal cargo, in addition to proteomic analysis [177], nucleic acids (mainly non-coding RNAs) play a leading role as biomarkers, having the advantage, compared to proteins, of the high sensitivity of the current detection systems, based on the polymerase chain reaction (PCR). Regarding protein biomarkers, the growth factor receptor EGFRvIII (a variant form of EGFR) stands out among the glioma-specific oncogenes; it is one of the most studied tumor-specific proteins, since the mutant receptor is present in EVs secreted by glioma cells [98,178]. Interestingly, the serum levels of EGFRvIII can be evaluated in the exosomal fraction, with non-invasive methods, but with high sensitivity and specificity [179].

Other biomarkers of considerable relevance in the early diagnosis of GBM are the mutated mRNA encoding the isocitrate dehydrogenase 1 (IDH1) enzyme and a group of miRNAs, among which are miR-21, which are present in glioma-derived EVs [180] and involved in the vascular proliferation of glioma. Furthermore, in a mouse GBM model, high levels of dynamin-3 (DNM3) and p65, and a decreased expression of p53, were observed in both brain and blood exosomes. This observation suggests that these proteins might be used as exosome-derived biomarkers in GBM [181], which both brain and blood exosomes were found to express.

Among the molecular targets which are studied, there are also other epigenetic factors that guide cancer development and therapeutic response. Therefore, the dysfunction of molecules with epigenetic roles has been widely explored as a potential target for the treatment of GBM. This latter cancer disease is one of the most studied tumors and has also been found to be regulated by the expression of histone genes. In some studies, it has been shown that in more than 80% of GBM cases, the H3K27M mutation is present in histones H3.1 and H3.3 [182]. The H3K27M mutation refers to the replacement of the lysine at position 27 with a methionine, and this causes a decrease in the trimethylation of histone H3. This mutation induces the deactivation of polycomb repressive complex 2 (PRC2) methyltransferase, thus promoting altered gene expression and further glioma progression [183].

In addition, the possibility has been suggested to use CRISPR/Cas9 technology which uses gene editing to treat pathologies in which there are genetic mutations, or for the expression of antigens, antibodies, or receptors as immunotherapy. Furthermore, some studies have used CRISPR/Cas9 to create in vitro models of gliomas to study tumor progression or to isolate possible biomarkers [184].

## 10. Therapies for GBM

Today’s standard treatment regimens include initial surgical resection of a large portion of the tumor mass, followed by radiation therapy, in combination with chemotherapy. Among the drugs used to treat GBM are temozolomide (TMZ), carmustine, bevacizumab, vorinostat, olaparib, lomustine, and valproic acid [185].

Radiotherapy is based on the effects of ionizing radiation on tumor cells. It indeed causes DNA damage. Radiotherapy together with temozolomide allows to obtain 9–20 months of survival in elderly patients (compared to the 6–8 months with standard radiotherapy) [185]. However, further research is needed to adapt the fractionation regimen and increase the survival rate and quality of life of patients.

Brachytherapy uses the radioactive isotopes I-125 and Ir-192 to deliver ionizing radiation into the tumor. Ir-192 is used in high-dose brachytherapy and is removed after a certain period. I-125 is mainly used for low-dose brachytherapy, and the introduced capsules often remain in the body, as it seems that they do not cause significant side effects. Most importantly, brachytherapy allows a localized action that can reduce the rate of tumor recurrence. Actually, however, some patients, treated with too high doses, underwent radionecrosis. In some studies, high-dose brachytherapy treatment in combination with surgery or external radiotherapy has been evaluated [186]. In this study, it was seen that this therapy allowed a greater patient survival in the absence of tumor progression; moreover, survival significantly increased also for inoperable patients [186].

Radiosurgery seems to have a significant efficacy in tumor progression and tumor recurrence, with an average survival rate of about 9 months. A better survival rate has been observed in patients treated with both radiosurgery and bevacizumab, as the tumor growth is also limited due to inhibition of angiogenesis [187].

Despite cumulative progress in diagnostic methods and multidisciplinary treatment, all patients with GBM ultimately experience inevitable tumor progression and death. Although with today’s treatment regimens an increase in the progression-free survival period has been obtained, overall survival remains unchanged [185]. This has been largely attributed to the gain of resistance by tumor cells that survived TMZ, through the increased expression of DNA O-6-methylguanine methyltransferase (MGMT): a mechanism called chemoresistance. 

In a recent study, the therapeutic approach was modified with the additional use of additional drugs, such as the new MGMT inhibitors, which were tested in the preclinical phase; however, these inhibitors present the great risk of inactivating DNA repair pathways in healthy cells [188]. A further approach involves the additional use of bevacizumab (in addition to TMZ), which only succeeded in prolonging the disease-free period, indicating that other resistance mechanisms are rapidly activated [189].

Therefore, in the current scenario and with the current weapons, the treatment of glioma has unfortunately become a challenge to gain as much time as possible for the patient, rather than an actual treatment method. The main obstacles faced by standard treatment are: (i) first of all, the invasive nature of the tumor itself eliminates any possibility of recovery, despite surgery; (ii) genetic heterogeneity at both the intertumoral and intratumoral levels determines the need to institute a much more varied therapy; (iii) the presence of the blood–brain barrier (BBB) hinders the systemic diffusion of drugs to tumor sites in the brain, thus slowing down the overall effectiveness of chemotherapy. Moreover, cancer cells tend to acquire drug resistance. Recently, it has been reported that chemoresistance is due, at least in part, to the high expression, in glioblastoma stem cells (GSCs), of members, such as ABCB4, of the ATP-binding cassette (ABC) transporters; moreover, ABCB4 can be transferred to the neighboring cells via EVs [190]. Recently, it has been also reported that, under the effects of EVs from GSCs, transformed astrocytes can deliver the AlkB Homolog 7 (ALKBH7) to the neighboring cells, thus causing temozolomide resistance [191].

In general, overexpression of P-glycoprotein (P-gp) is a major means by which tumor cells become resistant to multiple drugs, and this is a key factor in the acquisition of TMZ resistance by glioblastoma cells. Apparently, exosome-based drug delivery might bypass the P-gp efflux pump, thus reducing the required drug dosage [185]. In order to improve the therapeutic efficacy of a drug, at the same time reducing its off-target effects, it would be necessary that the drug molecules accumulate specifically in diseased areas, in a controlled manner, over a prolonged period of time.

It is also worth noting that the response to radiotherapy is not the same for all patients; this aspect depends on the fact that GBM is characterized by a high genetic and molecular variability. Thus, it is not possible to predict the response to therapy. There are also some forms of resistance with worse outcomes after radiotherapy [192,193]. Further studies on this therapeutic approach are therefore necessary to improve the prognosis and survival of patients. Radioresistance (RS) seems to be primarily associated with replication stress (RS) in GBM cells and probably involves the activation of processes responsible for stabilization of replication forks, as well as prevention of DNA damage. Among the parameters that indicate RS in glioblastoma stem cells (GSCs), increases in the replication protein A, in single-stranded DNA-binding proteins, and in various markers of DNA damage have been reported [194]. Anyway, radiation therapy is, at the moment, the most effective treatment for most primary tumors of the central nervous system. As mentioned, however, its effectiveness is limited by radiotherapy tolerance, with uninterrupted tumor growth after radiation and increased risk of metastatic progression; when this happens, of course, a change in the therapeutic protocol is required.

In general terms, radioresistance refers to the mechanism through which tumor cells are able to overcome the problems induced by radiotherapy, thanks to their high plasticity and variability, which allow modification of their microenvironment, metabolic reprogramming, regulation of gene transcription, DNA repairing, and overcoming the cell cycle checkpoints [185].

Some studies describe the existence of gliomas whose cells have self-renewal capabilities and present the typical molecular markers of GBM tumor stem cells: GSCs [185]. These cells express the CD133 protein (also known as prominin-1), which participates in GSC differentiation and self-renewal, two key processes in carcinogenesis, as well as in the development of resistance to radiotherapy [195]. Moreover, it has been shown that, although radiotherapy causes damage to tumor blood vessels, CD133-positive cells manage to survive, and cause tumor recurrence. Tissues taken from patients with glioma able to regrow express higher amounts of CD133 than tissues taken from recently diagnosed patients [195]. CD133-positive tumor cells are able, during the replicative checkpoint phase, to repair radiation-induced DNA damage, showing resistance to apoptosis [196].

Any organism is endowed with what has been called a chaperone system (CS) and that consists of molecular chaperones, co-chaperones, cofactors, and receptors. The canonical functions involve maintaining protein homeostasis, and, therefore, the CS maintains all enzymes and proteins in their native conformation, fundamental for their functions. In glioma development, molecular chaperones have a variety of roles, and it has been shown that Hsp60, Hsp70, and Hsp90 are transported by EVs produced by GBM tumor cells [196]. Hsp90 and Hsp47, for example, promote angiogenesis; while, Hsp70, Hsp40, and Hsp27 have a lengthening effect on the tumor cell survival [197]. Hsp90 is specifically involved in metabolic modifications and in transcription of genes involved in tumorigenesis and cancer progression. On the other hand, Hsp70 seems to be involved in glioblastoma resistance to radiotherapy because it can protect tumor cells from radiation [198].

In general, GSCs have special properties that allow them to counteract radiation-induced damage. Although the precise mechanisms of resistance are not yet fully known, it seems that an increase in DNA repair potential is involved. An essential part of cell response to DNA damage is the activation of cell cycle checkpoints, which induce temporary arrest of replication, in order to give cells enough time to correct the damage [185].

Homologous recombination repair (HRR), non-homologous end joining (NHEJ), and alternative NHEJs are the main pathways used by cells to repair radio-induced damage. The same processes are also involved in radioresistance promotion [199]. HRR occurs preferentially in the final part of the S phase of the cell cycle, in G2 and in M phase, when “sister” chromatids are present. On the other hand, NHEJ can be activated at any time of the cell cycle because it does not have a homologous DNA template; it is, indeed, the predominant repair pathway in G1 and G2 phases [200]. The phenomenon of radioresistance is especially noticed in the late S phase, probably because of the increased replication level homologous recombination [200].

In addition, overexpression of the epidermal growth factor receptor (EGFR) and its variant III (EGFRvIII) causes radioresistance in GBM, because of stimulation of both HRR and NHEJ. In particular, EGFRvIII induces the activation of the catalytic subunit of the DNA-dependent protein kinase (DNA-PKcs), which is involved in DSB repair [201].

In conclusion, therapeutic resistance represents a fundamental obstacle in GBM treatment. Moreover, multiple EV-mediated mechanisms of therapy resistance have been described for cancers as different as breast, prostate, lung, kidney, ovary, pancreas, stomach, and brain tumors. Now, as discussed above, EVs derived from tumor cells but also from the cells present in the cancer microenvironment transfer a variety of cargoes (mRNAs, miRNAs, lncRNAs, DNA, proteins, metabolites, and so on) that can have an impact on cells initially sensitive to therapeutic treatment, by inducing acquisition of a radio- or chemo-resistant phenotype [185].

Some studies have shown that the transfer of transcripts encoding DNA repairing enzymes, such as alkylpurine-DNA-N-glycosylase and O-6-methylguanine-DNA methyltransferase (MGMT), leads to an increase in repair capacity of DNA in recipient cells [202]. Moreover, it has been reported that regulation of the DNA repair pathways can be influenced by the EV-mediated secretion of miR-603; this miRNA, can indeed induce radioresistance, and even cross-resistance to alkylating agents [203].

## 11. Extracellular Vesicles: The Future of Glioma Therapy?

One of the techniques currently adopted to transport drugs to a tumor site in an efficient and selective manner consists in enclosing the molecules into a nanocarrier, which targets the tumor [204]. The nanocarrier systems include, in general, a specific surface factor that facilitates targeting to the tumor region. In the case of glioma therapy, nanocarrier systems must meet several criteria: (i) they have to be able to reach exclusively tumor cells, and, in particular, viable cells in the tumor mass, and not free cells already undergoing necrosis or apoptosis; (ii) they must target all the tumor cells, in spite of their biomolecular diversity, and heterogeneity; (iii) they should be able to cross the BBB, and to penetrate into the diseased regions of the brain; (iv) obviously, they should have a long-term half-life as well as a long-preserved delivery capacity in circulation, being also able to protect their cargo from degradation; (v) they should be easily detected and quantified in both tissues and body fluids, without major technical difficulties.

For example, interesting results in this field were obtained by designing a novel nanocarrier based on polylactic-co-glycolic acid (PLGA), linked to a specific drug, that aims at blocking the progression of glioma [205].

Since their discovery, drugs delivered by nanocarriers have produced promising results; however, the artificial nature of these nanocarrier systems presents significant problems related to their toxicity within the organism [206]. In order to ameliorate the drug targeting, without running into generalized toxicity problems, researchers are now analyzing the possibility to use as carriers more natural “vehicles”: EVs. Most of the properties required for a nanocarrier to reach the brain are actually satisfied by EVs that thus represent promising tools for studying and developing effective and personalized treatments for GBM. The promising EV properties include: (i) a small size, which may allow them to penetrate deeply into tissues, (ii) the ability to be immune-tolerated, (iii) a high circulation half-life and the ability to target specific subtypes of tumor cells, and, last but not least, (iv) they can easily cross the BBB. Moreover, EVs are able to transport both hydrophilic and hydrophobic drugs, since they are composed of a hydrophobic lipid bilayer enclosing a hydrophilic aqueous core layer. Thus, many researchers are studying the possibility to use in the future EVs as drug delivering systems to target brain tumors [127,174,207,208,209,210,211,212,213,214].

Several methods have been already developed to load drugs into EVs, but, in general, they can be described as either passive or active forms of encapsulation.

Interestingly, EVs also have the potential to be used in the development of anti-tumor vaccines. Tumor cells indeed express specific antigens that are transmitted to EVs derived from them. These EVs can activate DCs, which in turn can activate the anti-tumor capabilities of CD8+ T cells and NK cells. In one study, it was demonstrated that DCs, when incubated in the presence of tumor peptides, produced, in turn, EVs capable of activating the cytotoxic activity of CD8+ T cells, and suppressing tumor progression [215]. However, this application of EVs is still under test mainly in melanoma and non-small-cell lung cancer (NSCLC); it is possible that trials on glioma models will start in the near future. 

Finally, given the ability of tumor cell-derived EVs to play a key role in overall tumor progression, strategies to limit the secretion of such vesicles are now under investigation. For example, Munc 13-4, a Ca^2+^-dependent, soluble N-ethylmaleimide-sensitive factor attachment protein (SNAP) receptor, together with the Rab family proteins, have proven to be key regulators of high exosomal exocytosis. Therefore, their inhibition would allow targeted depletion and suppression of tumor secretion of exosomes, thus limiting cancer metastasis and angiogenesis [216]. Neutral sphingomyelinase 2 can also be considered a molecular target to block the release of exosomes [185].

A major challenge in glioma treatments is the difficulty of releasing the drug at the therapeutic dose only within the specific tumor tissue. Moreover, glioma cells as well as endothelial cells of the BBB express high amounts of low-density lipoprotein receptor-related protein-1 (LRP1), which mediates the transcytosis of multiple ligands across the BBB (such as lactoferrin, melanin, transferrin) [217]. 

Angiopep-2 (Ang2) is a peptide with a high affinity for LRP1 and has a high ability to penetrate the brain [217]. An Ang2 peptide-modified drug delivery system could increase the concentration of drug, which reaches the tumor target. However, it should be underlined that adequate quantities of drug cannot be administered to the patients with the LRP/Ang2 system, due to receptor saturation [218]. 

The trans-activator of transcription (TAT) peptide easily crosses the BBB and activates a non-saturable and receptor/transporter-independent process that allows entry into the dense tissues of the tumor. Therefore, engineered exosomes containing Ang2 and TAT synergistically allow both targeting to the center of the tumor and the concomitant overcoming of the saturation of the Ang2 receptor. This is why exosomes represent a new way to treat brain tumors [219]. The mechanism by which exosomes enter the BBB is not well known, but one of the factors involved appears to be the mannose 6-phosphate receptor which mediates transcytosis. Furthermore, some specific miRNAs (such as miRNA-181c) could alter the permeability of the BBB, by causing incorrect actin localization [220,221].

It is interesting to note that EVs not only show selectivity towards glioma cells but can also significantly enhance BBB permeation and tumor penetration. In fact, EVs loaded with doxorubicin, a P-gp substrate peptide, can be internalized more easily, evading the role of the efflux pump [219].

As previously discussed, EVs are taken up by cells through a wide range of both clathrin-dependent and clathrin-independent endocytosis pathways, including phagocytosis, micropinocytosis, caveolin-mediated uptake, and lipid raft [222]. Although the modified EVs target the brain tumor, it is also important to consider that the liver was found to exhibit a strong interception capacity for the modified EVs [223]. Therefore, it will be necessary, in the future, to develop engineered EVs that can avoid interception and elimination by liver or kidney.

We can, however, conclude that modified EVs are potentially a simple and effective nanoscale drug delivery tool in targeting chemotherapy drugs to the brain and, therefore, represent a promising approach for the future treatment of gliomas. 

## 12. Conclusions and Perspectives

In recent decades, an increasing number of brain tumor biological properties have been discovered, which have made it possible to highlight the molecular alterations that accompany cellular transformation towards an invasive phenotype. Despite significant advances in therapies, a large percentage of patients with GBM ultimately die, due to progression of the metastatic disease. It is, therefore, a key goal of glioma research to increase our understanding of invasive disease progression and its dynamics.

As herein discussed, EVs are involved in the ability of cells to migrate, but also in malignant transformation, neoangiogenesis, immunosuppression in the tumor microenvironment, immune tolerance, and resistance to treatment.

Among the most stimulating aspects of the study of EVs is the possibility of using them as diagnostic tumor markers, in addition to the fact that they might represent an alternative “vehicle” for drug administration, and effective treatment.

However, some questions regarding EV biogenesis and release still remain open. For example, it is not yet entirely clear why and how surrounding cells capture vesicles. Moreover, the difficulty in obtaining high penetration of the drug, at therapeutic doses, into the tumor site also remains a problem.

Given the essential role that intercellular communication plays between tumor cells and the cells of the microenvironment, it is of fundamental importance to clarify the aforementioned questions; a better understanding of these relationships, and of the “tumor ecosystem”, should indeed allow earlier diagnoses of brain cancer and the possibility to set up new therapeutic approaches for these tumors, which are still largely lethal.

## Data Availability

Not applicable.
